# Serum microRNAs associated with concussion in football players

**DOI:** 10.3389/fneur.2023.1155479

**Published:** 2023-04-18

**Authors:** Dorota Wyczechowska, Paul G. Harch, Shelly Mullenix, Erin S. Fannin, Brenda B. Chiappinelli, Duane Jeansonne, Adam Lassak, Nicolas G. Bazan, Francesca Peruzzi

**Affiliations:** ^1^Department of Interdisciplinary Oncology, Louisiana State University Health New Orleans, New Orleans, LA, United States; ^2^Department of Medicine, Section of Emergency Medicine, Louisiana State University Health Sciences Center, New Orleans, LA, United States; ^3^LSU Athletics, Louisiana State University, Baton Rouge, LA, United States; ^4^Neuroscience Center of Excellence, School of Medicine, Louisiana State University Health New Orleans, New Orleans, LA, United States; ^5^Department of Medicine, School of Medicine, Louisiana State University Health New Orleans, New Orleans, LA, United States

**Keywords:** miRNA - microRNA, concussion, mild traumatic brain injury (mTBI), sport injury, biomarkers

## Abstract

Mild Traumatic Brain Injury (mild TBI)/concussion is a common sports injury, especially common in football players. Repeated concussions are thought to lead to long-term brain damage including chronic traumatic encephalopathy (CTE). With the worldwide growing interest in studying sport-related concussion the search for biomarkers for early diagnosis and progression of neuronal injury has also became priority. MicroRNAs are short, non-coding RNAs that regulate gene expression post-transcriptionally. Due to their high stability in biological fluids, microRNAs can serve as biomarkers in a variety of diseases including pathologies of the nervous system. In this exploratory study, we have evaluated changes in the expression of selected serum miRNAs in collegiate football players obtained during a full practice and game season. We found a miRNA signature that can distinguish with good specificity and sensitivity players with concussions from non-concussed players. Furthermore, we found miRNAs associated with the acute phase (let-7c-5p, miR-16-5p, miR-181c-5p, miR-146a-5p, miR-154-5p, miR-431-5p, miR-151a-5p, miR-181d-5p, miR-487b-3p, miR-377-3p, miR-17-5p, miR-22-3p, and miR-126-5p) and those whose changes persist up to 4 months after concussion (miR-17-5p and miR-22-3p).

## Introduction

The goal of this study was to assess whether circulating microRNAs can serve as biomarkers of mild Traumatic Brain Injury in football players. Concussion, also called mild Traumatic Brain Injury (mTBI), is a complex condition induced by external mechanical forces on the brain. In general, mild TBI causes immediate neurological dysfunction, which is the result of microscopic physical injury to brain tissue (1). In most cases, although the neurological dysfunction resolves in a short period, the underlying physical injury to the brain does not (2). In up to 15% of individuals with mild TBI the immediate neurological dysfunction also does not resolve, or resolves only to recur within weeks and manifest as persistent cognitive dysfunction (3), the post-concussion syndrome (4). American football players are among the athletes at high risk for head injury. While advanced functional and structural MRI techniques may help in the diagnosis of mild TBI, they are not performed routinely to assess either clinical or subclinical TBI, allowing for repetitive TBI-and brain injuries-to accumulate (5). In addition, there is no quantitative method to assess the clinical severity of TBI or correlate tissue damage to the severity of the clinical syndrome. A non-invasive objective test able to identify and quantitate both symptomatic and asymptomatic brain injury would greatly improve the treatment and management programs. If early treatment were applied to clinical and even subclinical concussion, it would potentially limit the development of post-concussion syndrome or chronic TBI.

The dominant pathological injury in mild TBI is to the white matter (6). Axonal injury results from the rapid stretching of the axons, which then induce an uncontrolled flux of ions, increased intra-axonal Ca^2+^, release of glutamate and further depolarization of the neurons (1, 5, 6). Together with mechanical breakage of microtubules, the ionic changes result in interruption of axonal transport and accumulation of protein products. This accumulation defines the classic neuropathological phenotype of axonal swelling, which in the worst case, can lead to the disconnection of axons. Additional cascades of events may lead to apoptosis, mitochondrial dysfunction and inflammatory processes. Importantly, changes in microRNAs can reflect all those molecular and cellular events (7–10). Interestingly, repeated head trauma in boxers leads to a syndrome with clinical, pathological, and neurochemical similarities to Alzheimer's disease (AD) (11). This has also been recognized in professional football players, leading to chronic traumatic encephalopathy (CTE) (12–15).

MiRNAs are small 19–25 nucleotide, single-stranded, non-coding RNAs that regulate gene expression by inhibiting mRNA translation through incomplete base pairing (10, 16–18). Evidence shows that trauma can induce changes in the expression of miRNAs in the trauma-affected organ. In addition, miRNAs are abundant and relatively stable in the blood, in which changes in their expression can reflect pathological conditions. Changes in plasma or serum miRNAs have been associated with a variety of diseases, including animal (19) and human severe (20–25) and sport-related TBI (26–30). A recent systematic review of salivary miRNA in acute mTBI and persistent post-concussion syndrome revealed 14 miRNAs with consistent directional change after mTBI (31). One of the reviewed studies (32) developed a model combining symptoms and neurocognitive measures with miRNAs to aid with the diagnosis of mTBI, however, to date there are no biomarkers by themselves that identify or track acute mild TBI. Temporal assessment of plasma miRNA levels has been evaluated in male and female amateur football players, and it was found that circulating levels of 18 miRNA were deregulated in the acute phase of concussion compared to pre-concussion baseline (33). Our laboratory has developed a protocol to study miRNAs in plasma/serum and cerebrospinal fluid (CSF) as biomarkers for HIV-associated neurocognitive disorders (34–36). In the present study, we have evaluated serum miRNA expression from collegiate football players with and without concussion and found seven miRNAs associated with acute concussion. Furthermore, we have identified miRNAs associated with the acute phase post-concussion and miRNAs whose dysregulation persists when the players return to play about 3 weeks after concussion, as well as up to the end of season (about 4 months), potentially indicating neuronal injury even after clinical recovery.

## Materials and methods

This study was approved by LSUHSC-NO Institutional Review Board. All subjects from a college football team were informed of the study during an initial meeting with the athletic director and players. Written informed consent was obtained from all subjects before the beginning of practice season and prior to enrollment in the study. Subjects also filled out a preseason questionnaire that assessed demographic data, health and concussion history, concussion symptoms, and sleep patterns.

### Inclusion criteria

Subjects had to pass the team's history and physical examination, be a member of the 2014–2015 college football team, male, and 18–22 years of age.

### Exclusion criteria

Voluntary refusal to participate in the study.

### Protocol

When a significant impact to a player's head was witnessed by training staff, personnel, or team doctors, medical service had to be provided to a player on the field, a teammate reports another player's symptoms, signs or playing dysfunction, or a player requests an evaluation, the player is immediately evaluated by an athletic trainer and then a team physician on the sideline of the field. The player is taken to the athletic training room and completes the Graded Symptom Checklist which is a modified Sport Concussion Assessment Tool (SCAT-3) and the computer-based Immediate Post-concussion Assessment and Cognitive Testing (ImPACT) tests. The physician then makes the determination of a diagnosis of acute concussion based on a combination of history, physical exam, and these tests. If acute concussion is diagnosed the player is placed into the Athletic Training Concussion Management Protocol, which is based on CDC and NCAA guidelines and policies, removed from the field and observed/examined the rest of the day. Post-concussive care documentation is given to family or the player's roommate, and he is re-examined the following morning and daily by the athletic trainer and/or physician with completion of a symptom checklist. When symptoms have cleared, the player begins a graded exercise program before full return to play. They are returned to play when they are asymptomatic and have resolved any neurological exam abnormalities.

Upon diagnosis of concussion the player was immediately matched by position, weight, height, and concussion history (based on the questionnaires) to a control player. The control player was allowed to continue practice or play. Blood was drawn from the concussed player within 1 h of the injury and the matched control player at the end of that day's practice or game, usually within 2 h of the concussion. Additional blood samples were obtained from both concussed and control players at 18 h post-injury. The entire enrolled study population was again sampled at the end of the season. The concussed and control players' blood were collected in multiple tubes, labeled, and refrigerated until the end of that day's practice or game, then transported at 4°C in an iced biohazard cooler using a private courier to the LSUHSC-NO Neuroscience Center of Excellence. At the Center samples were immediately centrifuged at 3,500 rpm for 30 min to separate the serum. The serum was separated from the pellet and the sample stored at −80°C for later miRNA analysis.

### MiRNA selection

A total of 70 miRNAs were selected for analysis in this study based on the following criteria: these miRNAs are (1) enriched in the brain and/or have a validated function in the brain, particularly axonal and synaptic functions; (2) involved in neuroprotection, particularly after injury; and (3) associated with neurocognitive impairments and/or in non-sport related TBI. 17 out of the 70 profiled miRNAs were discarded because these were not detected in most or all of the samples. The resulting 50 miRNAs were subjected to further analysis (Table 1).

**Table 1:** List of miRNAs subjected to statistical analysis.

hsa-let-7b-3p	hsa-miR-210-3p
hsa-let-7b-5p	hsa-miR-21-5p
hsa-let-7c-5p	hsa-miR-221-3p
hsa-let-7d-3p	hsa-miR-222-3p
hsa-let-7d-5p	hsa-miR-22-3p
hsa-miR-106b-5p	hsa-miR-22-5p
hsa-miR-126-5p	hsa-miR-23a-3p
hsa-miR-127-3p	hsa-miR-23b-3p
hsa-miR-132-3p	hsa-miR-26a-5p
hsa-miR-133b	hsa-miR-29a-3p
hsa-miR-134-5p	hsa-miR-30b-5p
hsa-miR-143-3p	hsa-miR-320a
hsa-miR-146a-5p	hsa-miR-337-3p
hsa-miR-151a-5p	hsa-miR-338-3p
hsa-miR-16-5p	hsa-miR-374b-5p
hsa-miR-17-5p	hsa-miR-376a-3p
hsa-miR-181a-5p	hsa-miR-377-3p
hsa-miR-18a-3p	hsa-miR-431-5p
hsa-miR-18a-5p	hsa-miR-451a
hsa-miR-19a-3p	hsa-miR-487b
hsa-miR-19b-3p	hsa-miR-495-3p
hsa-miR-194-5p	hsa-miR-532-3p
hsa-miR-197-3p	hsa-miR-543
hsa-miR-200a-3p	hsa-miR-744-5p
hsa-miR-20a-5p	hsa-miR-92a-3p

### RNA extraction, quality control, and miRNA profiling

RNA extraction and miRNA profiling were performed as previously reported (34–36). RNA was obtained from 200 μl of serum using the miRCURY RNA extraction kit (Qiagen, Woburn, MA). To increase the RNA recovery, 1 μg of MS2 carrier RNA was added to each plasma sample. 8 μl of total RNA was subjected to retro-transcription using the Universal cDNA synthesis kit (Qiagen, Woburn, MA), followed by RT-qPCR using miRNA LNA primer sets (Qiagen, Woburn, MA). RT-qPCR was carried out in duplicate on a Roche LightCycler 480 Real-Time PCR System according to the Qiagen recommended protocol. Cycling conditions were as follows: 95°C for 10 min, 40 cycles of 15 s at 95°C, and 60 s at 60°C. Fluorescent data were converted into cycle threshold (Ct) measurements by the Roche LyghtCycler system software (Version 1.5; Roche). Quantification using 2nd derivative maximum was further calculated with Roche Lightcycler 480 software. qPCR data were analyzed in GenEx Professional 5 software (MultiD Analyses AB, Goteborg, Sweden). Degree of hemolysis was determined as the difference in Ct of miR-23a-3p (a miRNA not affected by hemolysis) and miR-451a (an indicator of hemolysis); this calculation was performed in GenEx. The amount of target miRNAs was normalized relative to the amount of miR-23a-3p reference gene, as determined by GeNorm, an application of GenEx software. MiRNA pair analysis was performed following a method published by Sheinerman et al. (37–39) and utilized previously by us (35, 36).

### Statistics

Statistical calculations were performed in GenEx Professional software. Mann-Whitney tests were two-sided and set at 5% level and 95% confidence intervals (CIs). Bonferroni correction was applied to determine statistically significant miRNA and miRNA pairs (*p* < 0.0001). The potential of miRNA and miRNA pairs for use in the diagnosis of concussion was assessed by estimating sensitivity and specificity based on an ROC analysis using GraphPad Prism 9.

## Results

### Study subjects

A total of 57 players from the 2014 football season (August through December) were enrolled and sampled. Ten sustained concussions and they were matched to 11 non-concussed control players (one control player was matched twice and had blood samples drawn separately for each of his matched concussed players) (Tables 2, 3). These 21 players were included in all data analyses. The average age of the 21 participants was 20 (range 18–23). Fourteen players (67%) were African-American, four (19%) were Caucasian, two (9%) were of mixed race, and one (5%) was Pacific Islander. Eight players (38%) noted having a prior concussion. Six players who had prior concussions stated that football was the cause. The other two forms were left blank. Two players noted having more than one prior concussion. One player noted a history of depression. Fourteen players (67%) stated they slept between 4 and 8 h per night. Of the 10 concussed players six (60%) had previous concussions and six were linemen, three of whom had previous concussions. Only two (18%) of the control players had previous concussions.

**Table 2:** Player demographics.

**Subjects**	**Non- Concussed**	**%**	**Concussed**	**%**
Number	11		10	
Mean age	19		20	
African American	8	73	6	60
Caucasian	3	27	1	10
Pacific Islander	0	0	1	10
Mixed race	0	0	2	20
Previous concussion	2	18	6	60
Depression	0	0	1	10
Current smoker	1	9	1	10
Past smoker	6	55	6	60

Demographics for the concussed and non-concussed players, provided by the self-reported player questionnaires.

**Table 3:** List of players and the dates of blood withdrawal at the time of concussion, after 18 hrs and at the end of the season.

**Concussed**							
	**Date of Concussion**	**Position**	**date 2h**	**date 18h**	**date ret play**	**End of season**	**# days from conc**
1	8/5	DT	8/5	8/6	8/23	12/22	139
2	8/8	LB	8/8	8/9	8/23	12/22	136
3	8/8	WR	8/8	8/9	8/16	12/22	136
4	8/9	OL		8/10	8/13	12/22	135
5	8/9	OL	8/9		8/23	12/22	135
6	8/21	C	8/21	8/22			
7	9/23	DT	9/23	9/24		12/22	91
8	9/27	FB	9/27	9/28		12/22	87
9	10/11	DE	10/11	10/12		12/22	73
10	11/19	S	11/19	11/20		12/22	33
							
**Controls**							
	**Date**	**Position**	**date 2h**	**date 18h**		**End of season**	
1	8/5	DE	8/5	8/6		12/22	
2	8/8	LB	8/8	8/8		12/22	
3	8/8	WR	8/8	8/9		12/22	
4	8/9	OL	8/9	8/10		12/22	
5	8/9	C	8/9			12/22	
6	8/21	C	8/21	8/23		12/22	
7	9/14	S	9/14	9/14			
8	9/23	DE	9/23	9/24		12/22	
9	9/27	FB	9/27				
10	10/11	DT	10/11	10/12		12/22	
11	11/19	S	11/19	11/20		12/22	

For 5 players, blood was collected when they returned to play. The number of days after concussion by the end of the season is reported, as well as the player position: DT, defensive tackle; C, center; FB, fullback; WR, wide receiver; TE, tight end; OL, left and right guard; OG, offensive guard; OT, offensive tackle; DE, defensive end; LB, linebacker; S, safety.

### MiRNAs associated with concussion

We profiled serum miRNAs from the 10 concussed and 11 non-concussed control players. Blood was collected for each player at 2 and 18 h post-concussion, as well as at the end of the season (average 106.4 days, Table 3). All players in the concussed group had their blood drawn at the three timepoints, except for three players, each missing either a 2, 18 h, or end of season timepoint. One sample in the 18 h concussed group was discarded because of poor RNA quality. In the control group we did not have the 18 h blood draw for two players and the end of the season blood draw for two players.

**Figure 1:**
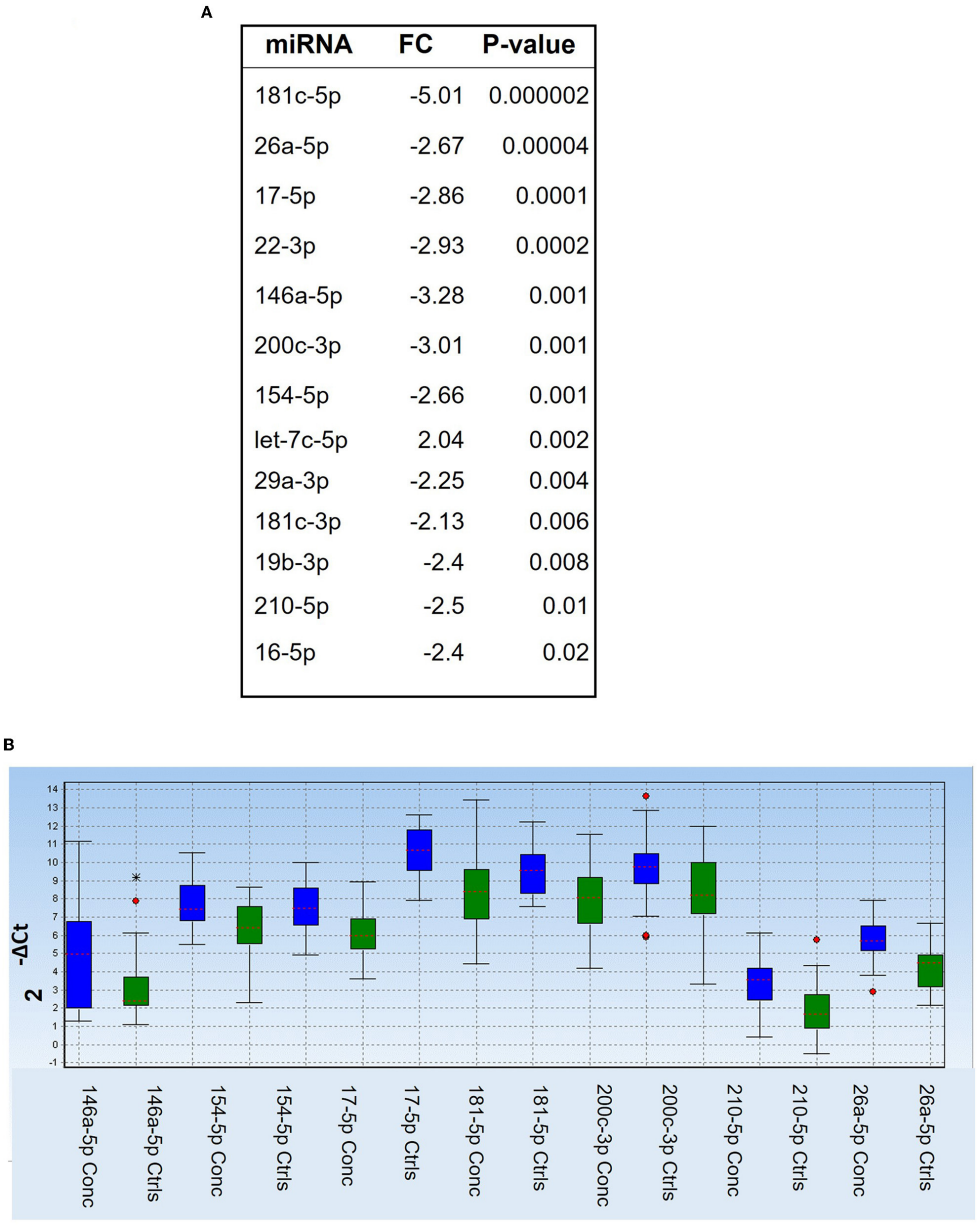
Differentially regulated miRNAs in concussed players compared to controls. **(A)** List of differentially regulated miRNAs at all combined timepoints, as determined by Mann-Whitney test. FC: fold change (2^−ΔΔCt^). *P*-values are shown in the right column. **(B)** Box plot showing relative expression (2^−ΔCt^) of the selected miRNAs in the two groups of concussed (*n* = 27; blue) and control players (*n* = 29, green). Each box shows the distribution of the measured miRNA value across the samples. The red dotted line in each colored box represents the median value. Red dot stars identify outliers and extra outliers, respectively.

**Figure 2:**
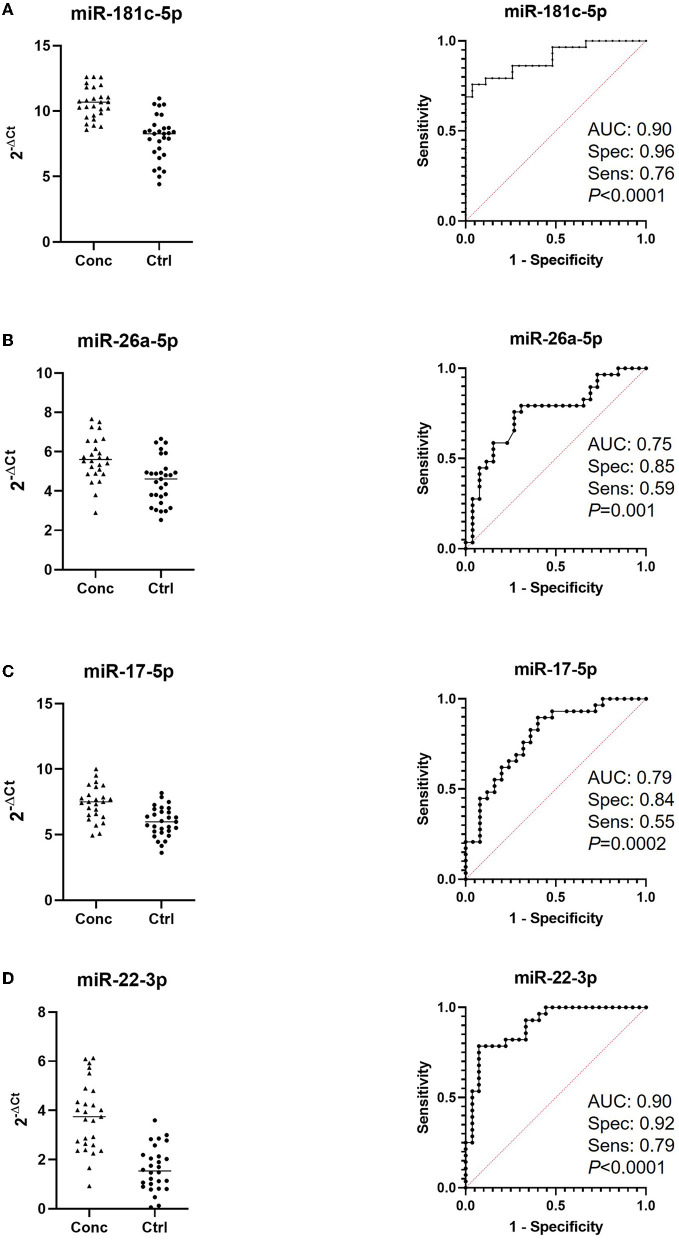
Receiver-Operating Characteristic (ROC) analysis of miRNAs discriminating concussed players from controls. Left panels indicate relative expression of the indicated miRNAs in concussed and control players. Note that higher Cts indicate lower expression. Area under the curve (AUC), sensitivity and specificity are calculated for the cutoff point and are indicated in the ROC graphs together with the *p*-values (right panels); **(A)** miR-181c-5p (concussed *n* = 27, controls *n* = 29); **(B)** miRNA-26a-5p (concussed *n* = 26, controls *n* = 29); **(C)** miRNA-17-5p (concussed *n* = 25, controls *n* = 29); **(D)** miR-22-3p (concussed *n* = 27, controls *n* = 28).

We first compared the combined 2, 18 h and end of season timepoints from concussed players (*n* = 27 samples with controls (*n* = 29 samples) and found one miRNA, Let-7c-5p, upregulated in concussed players, while 12 miRNAs were downregulated (miR-181c-5p, miR-146a-5p, miR-200c-3p, miR-22-3p, miR-17-5p, miR-26a-5p, miR-154-5p, miR-210-5p, miR-19b-3p, miR-16-5p, miR-29a-3p, and miR-181c-3p) (Figure 1A). Figure 1B shows relative expression of the most differentially regulated miRNAs in the two groups. The predictive potential of the four miRNAs that had the lowest *p*-value (miR-181c-5p, miR-26a-5p, miR-17-5p, and miR-22-3p) was determined through Receiver Operator Characteristic (ROC) analysis, and the results are shown in Figure 2 (right panels) in which the area under the curve (AUC), specificity, sensitivity, and *p*-values are also indicated. Relative expression of each of these four miRNAs in concussed and control players is also indicated in Figure 2 (left panels). Note that the relative miRNA expression (calculated as 2^−ΔCt^) in Figure 2 correlates with normalized cycle thresholds, Cts; therefore, higher numbers in the y-axis mean less expressed miRNA.

**Figure 3:**
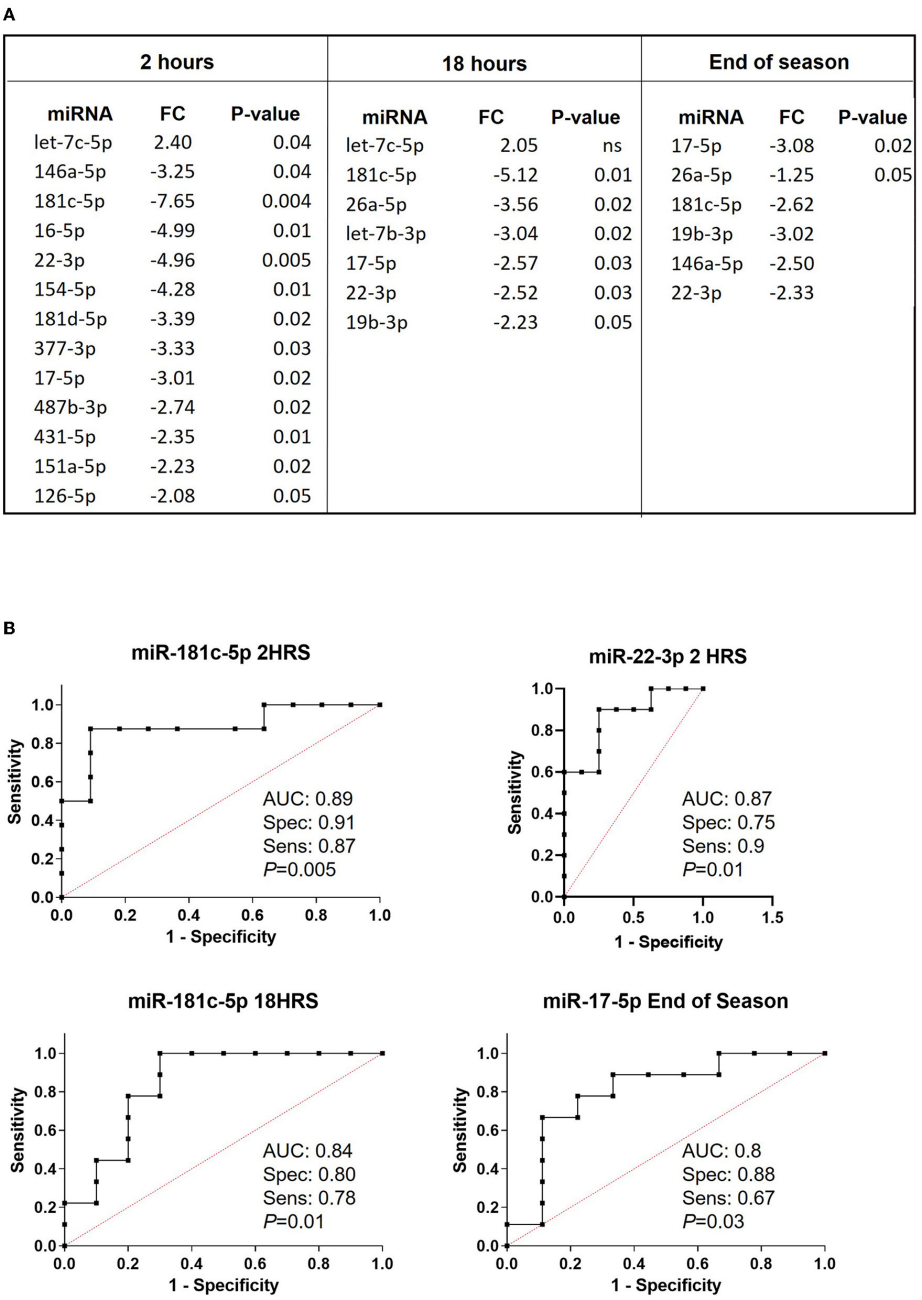
Relative expression of miRNAs discriminating concussed players from controls at different time points after concussion and at the end of the season. **(A)** List of differentially regulated miRNAs in concussed players vs. controls at 2 h (concussed *n* = 9, controls *n* = 11), 18 h (concussed *n* = 9, controls *n* = 9) after concussion, and at the end of the season (concussed *n* = 9, controls *n* = 9). FC: fold change expressed as 2^−ΔΔCt^. *P*-values are reported in each table. Ns: non-statistically significant. **(B)** ROC analysis graphs for the most dysregulated miRNA with the lowest *p*-value at each timepoint. AUC, sensitivity, specificity and exact *p*-values are indicated in the graphs.

Next, we compared each timepoint (2, 18 h, and end of the season) from concussed players to matching timepoints from controls. Results in Figure 3A indicate the fold change in miRNA expression in concussed players compared to controls at 2 h, 18 h, and at the End of the Season. Three miRNAs, miR-181c-5p, miR-22-3p and miR-17-5p were differentially regulated at all the time-points. Let-7c-5p was the only miRNA upregulated within 2 h from concussion and remained upregulated after 18 h, although the difference in the expression was not statistically significant at this timepoint. Conversely, the 2-3-fold decreased expression of miR-17-5p was statistically significant at each timepoint. Two of the most downregulated miRNAs at the 2 h timepoint, miR-181c-5p and miR-22-3p were also downregulated at 18 h, as well as at the End of the Season, although the downregulation was not statistically significant at the latter time-point. Unique miRNAs were differentially regulated at the 2 h time-point (miR-16-5p, miR-154-5p, miR-431-5p, miR-151a-5p, miR-181d-5p, miR-487b-3p, miR-377-3p, and miR-126-5p), and 18 h timepoint (Let-7b-3p). MiR-26a-5p was significantly differentially downregulated at the 18 h and End of Season timepoints, while miR-19b-3p was significantly downregulated at 18 h and not significantly at End of Season. Figure 3B shows plots of ROC analyses performed for the most dysregulated miRNAs with the lowest *p*-value at each timepoint.

#### Differentially regulated miRNA pairs

**Figure 4:**
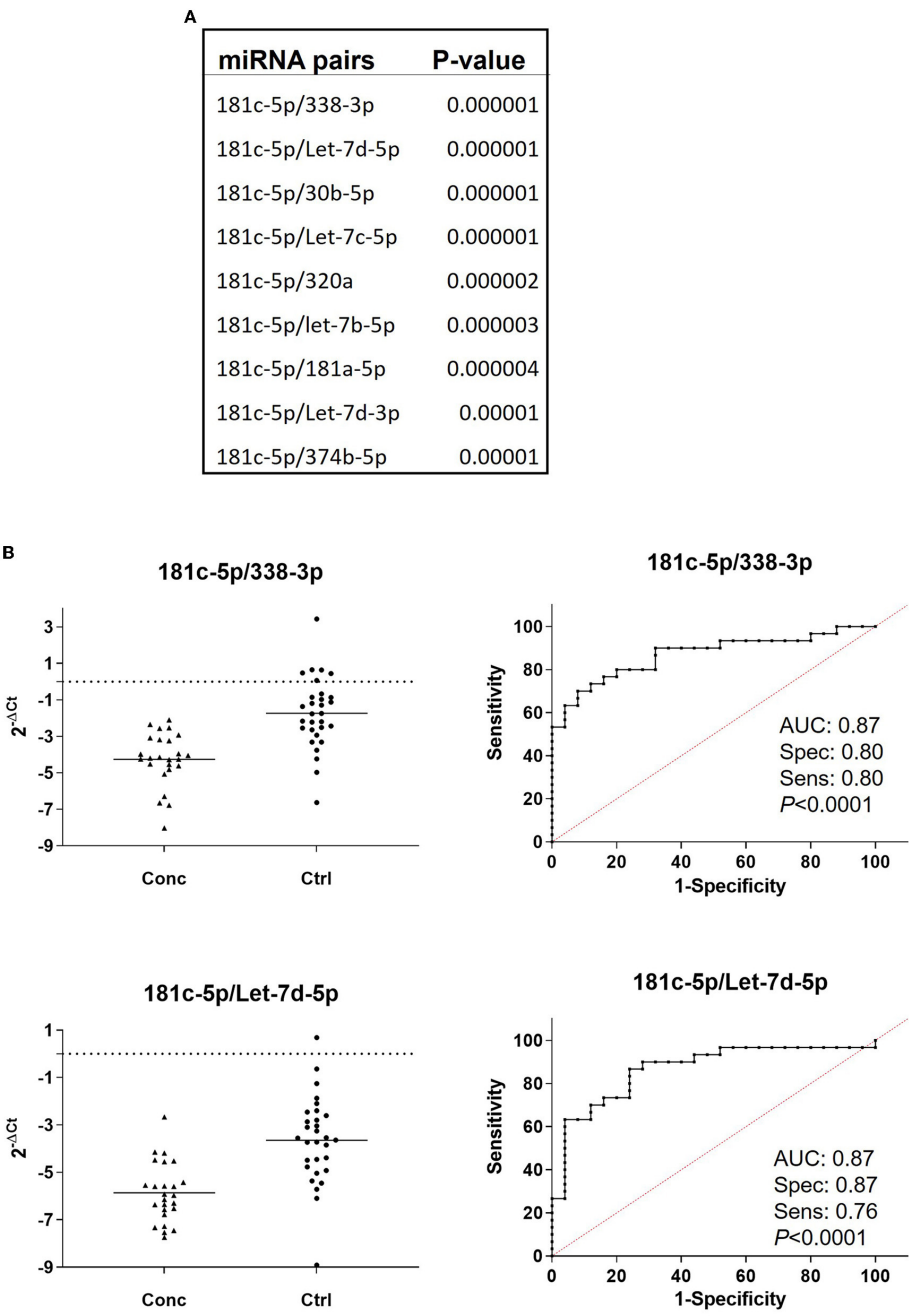
miRNA pair analysis increases sensitivity and specificity of miRNA biomarkers. **(A)** List of the miRNA pairs that best discriminate concussed players from controls at all the combined time points (concussed *n* = 25, controls *n* = 27). *P*-values are indicated. **(B)** Relative expression and ROC curves of the top ranked miRNA pairs, 181c-5p/miR-338-3p and miR-181c-5p/Let-7d-5p. AUC specificity and sensitivity are reported under the graph.

**Figure 5:**
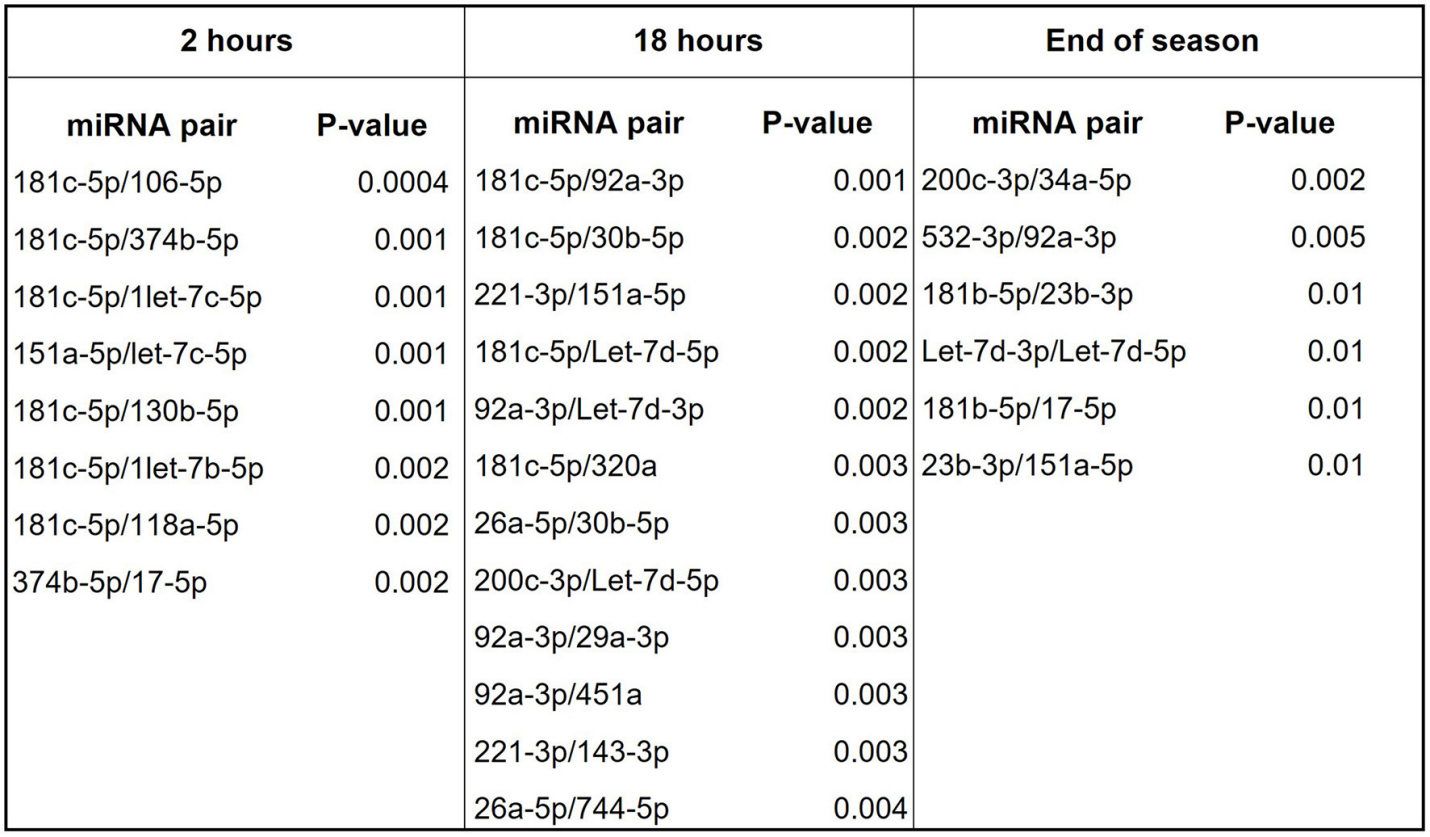
miRNA pairs discriminating concussed players from controls at various points during game season. List of miRNA pairs that best discriminate concussed players from controls at the indicated timepoints.

We then evaluated the diagnostic potential of the combination of miRNAs through the analysis of miRNA pairs (36, 40–42). Figure 4A shows a list of 9 miRNA pairs that better differentiated concussed players from controls (*p* ≤ 0.00001) at the combined time points. The pair miR-181c-5p/miR-338-3p was the best in differentiating with good sensitivity and specificity (Figure 4B) concussed players from controls. The other 8 miRNA pairs had similar *p*-value and comparable specificity and sensitivity; therefore, Figure 4B shows the ROC analysis only for the best overall pair (miR-181c-5p/miR-338-3p) and the best pair of the remaining 8 pairs (miR-181c-5p/let-7d-5p). Next, we compared miRNA pairs of controls and concussed players at each timepoint (Figure 5). Overall, miR-181c-5p was the most represented miR at the earlier timepoints of 2 and 18 h after brain injury. Interestingly, members of the Let-7 family, and the cluster miR-17-92, were represented in different pair combinations at each timepoint.

#### Downregulation of selected miRNAs is still observed in players who came back to play after concussion

**Figure 6:**
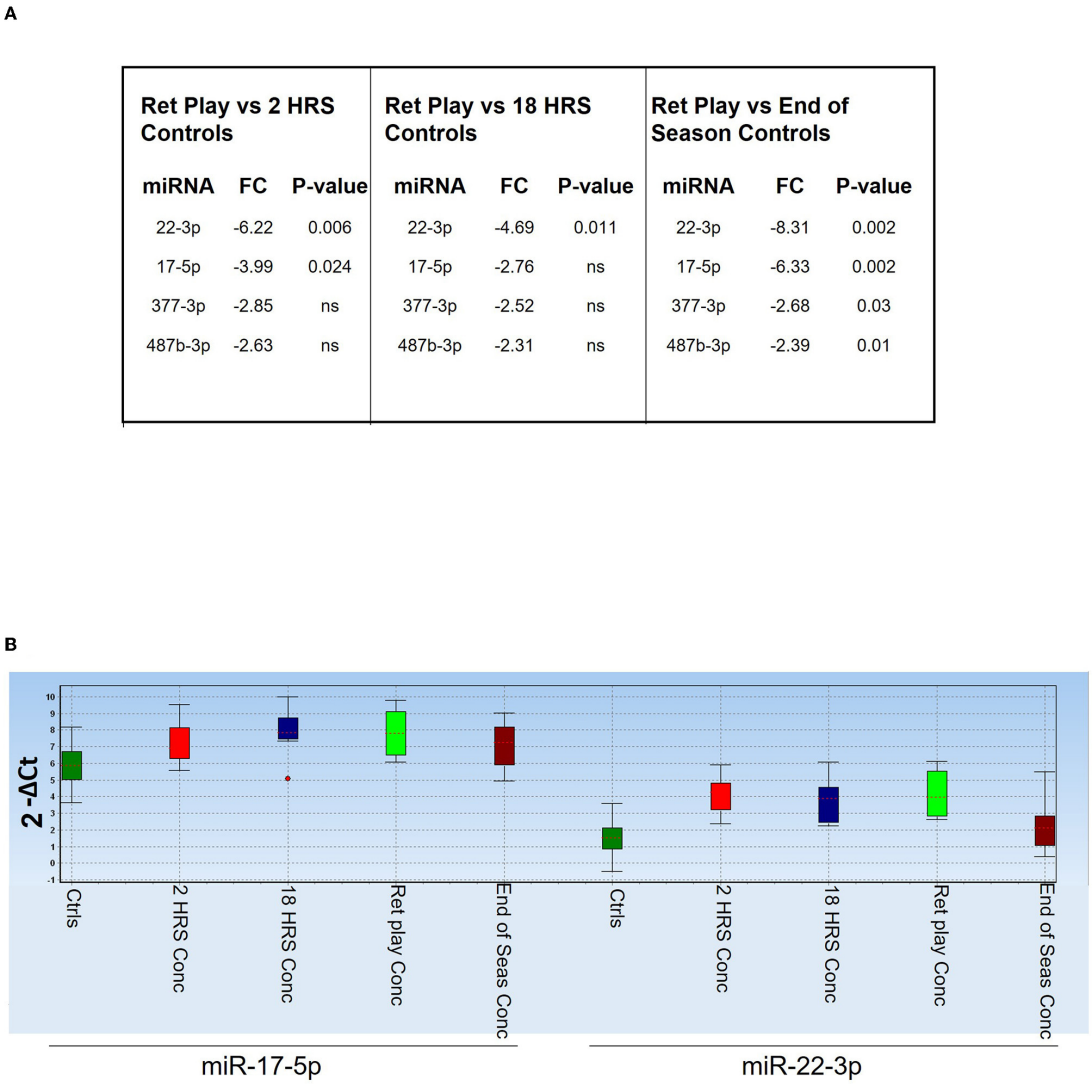
Diagnostic value of miR-17-5p and miR-22-3p. **(A)** Table showing miRNAs that discriminated players returned to the field after concussion (*n* = 5) from controls [overall (*n* = 29), 2 h (*n* = 11), 18 h (*n* = 9), and End of the Season (*n* = 9)]. *P*-values are indicated; ns means non-statistically significant. FC: fold change expressed as 2^−ΔΔCt^. **(B)** Expression levels of miR-17-5p and miR-22-3p in the indicated groups of players, measured as 2^−ΔCt^. The dot indicates an outlier.

We analyzed expression of selected miRNAs identified in the above analyses (miR-133b, 143-3p, 146a-5p, 16-5p, 17-5p, 19b-3p, 221-3p, 22-3p, 26a-5p, 29a-3p, 338-3p, 377-3p, 487b-3p, Let-7c-5p) in five players who returned to play after concussion compared to controls (Figure 6). We found that at each timepoint miR-22-3p and miR-17-5p still discriminated players who returned to play after concussion from controls (Figure 6A), suggesting that changes in circulating miRNA biomarkers could persist even after recovery from the symptoms of concussion. Figure 6B shows the relative expression of miR-17-5p and miR-22-3p in the indicated groups and highlights their sustained downregulation (higher Ct values) throughout the game season compared to controls.

#### Dysregulation of miR-17-5p in players with potentially undiagnosed concussion

**Figure 7:**
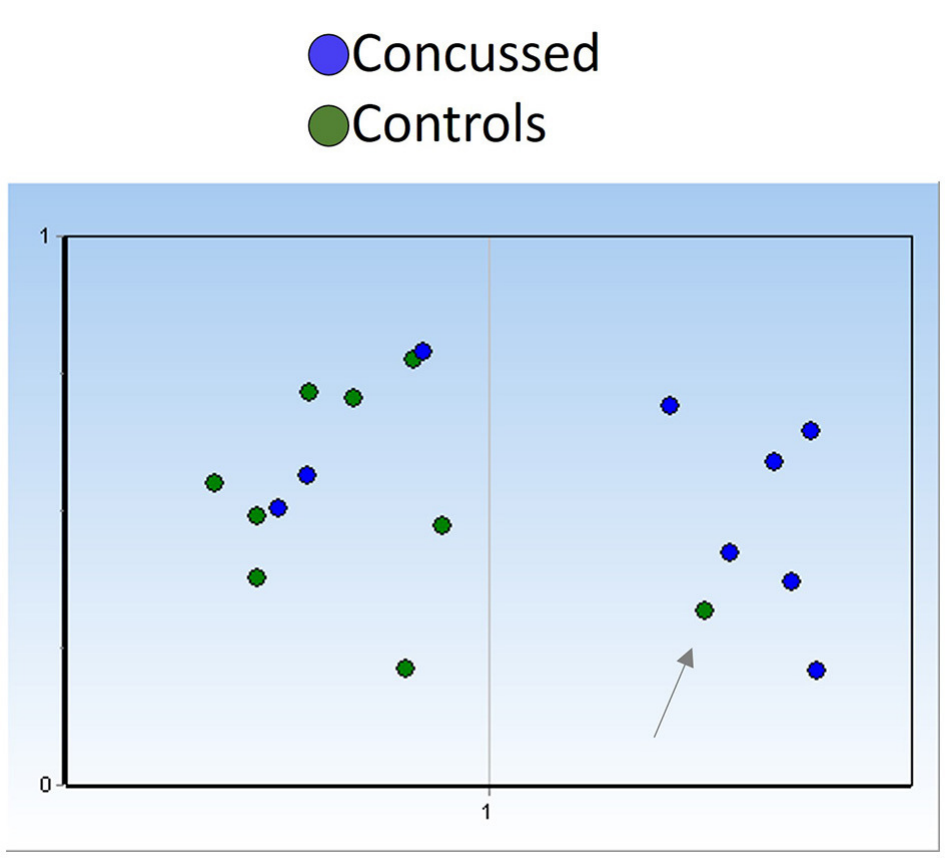
miR-17-5p could detect undiagnosed concussion and/or indicate restored neuronal fitness after concussion. Unsupervised clustering (Kohonen self-organizing map) of serum samples based on miR-17-5p expression at the end of season timepoint (controls *n* = 9; concussed *n* = 9) indicates the presence of three players who had concussion (blue dots) in group 1, otherwise constituted by control players green dots, suggesting neuronal fitness recovery. The presence of one control sample (pointed by an arrow) within the group of concussed players may indicate an undiagnosed brain injury in this player. Self-organizing map (SOM) was fixed automatically at 1 (*y*-axis), and number of groups are indicated in the *x*-axis.

Based on miR-17-5p expression levels at the end of the season, we generated two unsupervised clusters of samples using the Kohonen self-organizing map available in GenEx software and position-matched players (9 concussed and 9 controls). In general, control players mapped within group 1 (Figure 7, green dots) and concussed players clustered with group 2 (blue dots). Interestingly, only three out of 9 players who returned to play after concussion clustered with the group of control players (group 1), perhaps indicating a restored neuronal fitness after the injury. On the other hand, one sample in the control group mapped with group 2 (Figure 7, right panel), and this may be indicative of a non-diagnosed concussion.

## Discussion

In this exploratory study, we have utilized serum samples collected over a period of 4 months during a college practice and game season and determined the relative expression of selected miRNAs in concussed players vs. matched (same position in the field) controls. Since physical exercise profoundly affects miRNA expression (43–45), we reasoned that the best control for the concussed players would be a player exposed to a similar physical exercise but without TBI. The serum in control players was obtained at the same time as the concussed players (Table 3). We analyzed 50 miRNAs and found that 13 miRNAs (Figure 1) discriminated concussed players from controls (at all combined timepoints) and four of them with very good sensitivity and specificity (Figure 2).

When we compared samples from concussed players and controls obtained at the same timepoint, we found 13 miRNAs that discriminated the two groups at 2 h, 7 miRNAs at 18 h, and 6 miRNAs at the end of the season (Figure 3A). Notably, Let-7c-5p was the only miRNA consistently upregulated significantly at 2h and insignificantly at 18 h after concussion. The most downregulated miRNA at the 2-h timepoint (fold change−7.65), miR-181c-5p, maintained more than a 5-fold reduction after 18 h from concussion and almost 3-fold at the end of the season. Interestingly, one study reported that miR-181c-5p was part of miRNA signature for primary blast-induced mild TBI evaluated in hair follicles (46). We also found that miR-17-5p was significantly downregulated near or >3-fold at every timepoint, including the end of the season. This is important, since miR-17-92 cluster facilitates neuronal differentiation and neuroprotection under neuroinflammatory conditions (47–49). Unlike Papa et al., which found miR-26a-5p upregulated after TBI (50), we found the same miRNA downregulated 18 h after concussion and at the end of the season (Figure 3). This discrepancy may originate from the different groups used as the control in the two studies: Papa used blood withdrawn at baseline from the same players, while we used blood from players exposed to the same training but without concussion.

The persistence of the changes in the expression of miR-17-5p and miR-22-3p to the end of the season may indicate long-lasting molecular changes even after full clinical recovery. Interestingly, plasma miR-26a-5p and miR-16-5p discriminated non-sport related TBI from healthy controls (24) perhaps confirming a common pattern of injury in sport and non-sport-induced TBI. Let-7c has been shown to be differentially regulated in cellular and animal models of brain injury (ischemia and or TBI) and have been proposed to have a neuroprotective function (51–55). Downregulation of miR-22-3p has been observed in a cell model of TBI (55) and in neurodegenerative disorders such as Huntington's disease and AD (7, 56, 57). Overexpression of miR-22-3p attenuated neuronal injury caused by TBI (55) and protected from cell death in models of neurodegeneration (33). Finally, changes in circulating levels of miR-17, miR-92a, and miR-106a have been associated with schizophrenia (58, 59). Remarkably, our data indicate miR-17-5p and miR-22-3p as diagnostic markers potentially able to discriminate persistence of neuronal damage even after physical recovery (Figure 6A). In addition, the sole expression of miR-17-5p was able to cluster controls and concussed players (Figure 7). The presence of a control sample within the group of concussed players may indicate an undiagnosed brain injury and the presence of concussed players in the control group may indicate healing of concussion.

MiR-19b-3p belongs to the miR-17-92 family of miRNAs, a cluster consisting of six miRNAs (miR-17, miR-18a, miR-19a, miR-20a, miR-19b-1, and miR-92a-1) that regulates neurogenesis and angiogenesis in the central nervous system during development and adulthood [reviewed in (60)]. MiR-92a-3p and miR-17-5p distinguished concussed players from controls with good *p*-values when paired with other miRNAs (Figure 5). Of interest, plasma miR-92a also differentiated non-sport-related TBI from healthy controls (24). The most downregulated miRNA, miR-181c-5p (Figure 3), is involved in neuroinflammatory responses in glial cells (61, 62) and it was part of a Extracellular vesicle (EV) miRNA cargo signature associated with TBI (63). The miRNA pair that overall discriminated concussed from control players was miR-181c-5p/miR-338-3p (Figure 4). Of interest, thalamic-enriched miR-338-3p is a key mediator of synaptic disruption in the auditory thalamocortical circuit and the pathogenic mechanisms underlying psychosis in a mouse model of 22q11.2 deletion syndrome and related cases of schizophrenia (64). The association between miR-338-3p expression and neurodegeneration has been additionally shown in sporadic amyotrophic lateral sclerosis patients (65) and prion induced neurodegeneration (66). The miRNA-pair analysis approach was first described by Sheinerman to investigate plasma miRNAs as potential biomarkers for mild cognitive disorders (37), and we have used this type of analysis for cerebrospinal fluid and circulating miRNAs as biomarkers of neurocognitive impairments in HIV-1 infection (35, 36). In this study, miRNA pair analysis confirmed the importance of miR-181c-5p expression combined with the expression levels of miR-338-3p or Let-7-5p in discriminating concussed from non-concussed players (Figure 4). Interestingly, and of considerable diagnostic interest in the long-term development of brain degeneration, was the observation of a selective increase of a neurodegeneration-associated Let-7c-5p (Figure 1A), a chromosome-21q21-encoded miRNA also known to be specifically upregulated in Downs syndrome (trisomy 21) (67, 68), AD (69, 70) and depression (71, 72). The selective upregulation of this specific miRNA may be related to and predictive for the initial triggering of brain damage and the onset of neurodegeneration characteristic of both acute and chronic brain injury as evidenced by TBI and AD.

A recent systematic review of salivary miRNA/TBI studies (31) is consistent with the results of our study. Eight of the nine reviewed studies contained exclusively mTBI subjects and seven of the nine involved acute mTBI. The findings varied widely, but despite the broad heterogeneity in TBI by sport, sex, age, and other factors the authors identified 14 miRNAs with consistent up or downregulation across the nine studies: let-7i-5p, miR-107, miR-135b-5p, miR-148a-3p, miR-20a-5p, miR-24-3p, miR-27b-3p, miR-29c-3p, miR-181a-5p, miR-182-5p, miR-26b-5p, miR-320c, miR-27a-5p, miR-7-1-3p. We found that 4 of the significantly regulated serum-sampled miRNAs in our study are in the same families as these 14: Let-7c-5p, miR-26a-5p, miR-29a-3p, and miR-181c-5p. In another review of 14 human TBI studies (20) 17 miRNAs were found commonly in saliva, blood, and cerebral spinal fluid. Six of these 17 were from miRNA families found in our study: Let-7, miR-16, miR-26b, miR-29a, miR-29c, miR-181a. In conclusion, the miRNAs detected in our concussion study overlap with the findings across multiple clinical studies that sampled different body fluids. In a longitudinal study, Shultz et al. have examined plasma miRNAs as biomarkers of concussion in amateur football players (both females and males) and found decreased expression of miR-27a and miR-221 levels that inversely correlated with concussion symptom severity (30). However, their use of each concussed player's pre-season blood sample as a control did not control for miRNA from cumulative musculo-skeletal injury. In our study, we did not have pre-season blood samples, but controlled for musculo-skeletal injury miRNA by using concurrent position matched player controls. As we screened for specific, miR-27a was not included in our selection; however, we found miR-221 downregulated in players who sustained concussion at the end of season time point compared to the same time point of controls, although the difference was not reaching statistical significance (*p* = 0.056; data not shown).

Limitations in our study pertain to the small number of subjects, the gender (all males) and race (mostly African American), the screening limited to specific miRNAs, and lack of correlation between miRNA expression and specific symptoms of concussion. Nevertheless, important strengths of this study include following the same team throughout an entire game season, having a narrow age range (18–22), collecting the end of season time point (as far as 4 months post-concussion), and collecting blood from controls that matches the same time point of concussed players.

In summary, we provide evidence of a serum miRNA signature of 13 miRNAs associated with concussion in football players. Of interest, this miRNA signature suggests long-lasting molecular changes potentially associated with pathological behaviors that could also be explored in follow-up longitudinal studies. The upregulation of the Let-7c-5p miRNA may be a useful biomarker related to and predictive of the initial triggering of brain damage and the onset of neurological deficits in acute and chronic neurodegeneration as evidenced by concussive brain injury, TBI, Down's syndrome, AD, and psychiatric disorders (67–72). At the same time, miR-17-5p and miR-22-3p may be useful biomarkers for persistent neuronal injury from mTBI.

## Data availability statement

The original contributions presented in the study are included in the article/supplementary material, further inquiries can be directed to the corresponding author.

## Ethics statement

The studies involving human participants were reviewed and approved by LSUHSC-NO Institutional Review Board. The patients/participants provided their written informed consent to participate in this study.

## Author contributions

PH and FP planned and designed the miRNA study. DW, DJ, and AL performed the miRNA experiments. SM, EF, and BC coordinated sample collections. NB supported the study and provided the serum samples. All authors contributed to the article and approved the submitted version.
